# Application of Sequential Extraction Using Pressurized Fluids to Obtain Compounds from *Pereskia aculeata* Leaves

**DOI:** 10.3390/plants14131956

**Published:** 2025-06-26

**Authors:** Fernanda Rengel dos Passos, Mônica Lady Fiorese, Edson Antonio da Silva, Oscar de Oliveira Santos Junior, Lúcio Cardozo-Filho, Camila da Silva

**Affiliations:** 1 Programa de Pós-Graduação em Engenharia Química, Departamento de Engenharia Química, Universidade Estadual de Maringá, Av. Colombo, 5790, Maringá 87020-900, PR, Brazil; frengel.passos@gmail.com (F.R.d.P.); lcfilho@uem.br (L.C.-F.); 2 Programa de Pós-Graduação em Engenharia Química, Universidade Estadual do Oeste do Paraná, Rua da Faculdade, 645, Toledo 85903-000, PR, Brazil; mlfiorese@gmail.com (M.L.F.); edsondeq@hotmail.com (E.A.d.S.); 3 Departamento de Química (DQI), Universidade Estadual de Maringá (UEM), Av. Colombo, Maringá 87020-900, PR, Brazil; oosjunior@uem.br; 4 Departamento de Tecnologia, Centro de Tecnologia, Universidade Estadual de Maringá, Av. Ângelo Moreira da Fonseca, 1800, Umuarama 87506-370, PR, Brazil

**Keywords:** ora-pro-nobis, phenolic compounds, antioxidant activity, supercritical fluid extraction, pressurized liquid extraction

## Abstract

The aim of this study was to use high-pressure extraction methods to obtain compounds of different classes from the leaves of *Pereskia aculeata* Mill. For this purpose, Supercritical Fluid Extraction (SFE) and Pressurized Liquid Extraction (PLE) were used. SFE was performed with *Pereskia aculeata* leaves to evaluate the application of propane and carbon dioxide as solvents, and the residual biomass from this stage was used in PLE with hydroethanolic solvent. The extracts were characterized in relation to the content of phenolic compounds, antioxidant potential and content of nonpolar compounds. In the first stage, despite the low yield (1.09–1.94%) compared to PLE (16.56–19.26%), the extracts presented a high content of lipophilic compounds (squalene, octacosanol, α-tocopherol and β-sitosterol) compared to the PLE technique. The sequential extraction process benefited the greater recovery of phenolic compounds and extracts with greater antioxidant potential. Caffeic and nicotinic acids were the major compounds identified in the phenolic profile. The processes applied did not influence the protein content of the final extraction residue, which was similar to that of the in natura leaf. The results and approach demonstrate that sequential extraction is an excellent alternative for the use of *Pereskia aculeata*, which allows for the production of extracts with varied composition and/or extracts with greater recovery of compounds available in the plant.

## 1. Introduction

The biorefinery concept is a promising strategy for industries due to the maximum use of raw materials. Thus, the extraction of active compounds from plant sources through the integration of industrial processes allows for the use of green solvents and new technologies. The use of sequential processes using different solvents can recover different classes of compounds from plant matrices and, as a result, some solvents become more attractive for the extraction process. The use of pressurized propane is justified due to the high yields and short extraction times, resulting in products of high quality originating from the extracted active compounds and antioxidant properties [1,2,3]. Additionally, the use of this solvent to obtain natural products is advantageous because it does not leave residues due to its rapid evaporation after extraction; thus, it has become an alternative green solvent for obtaining natural and edible products.

However, the use of propane requires special attention due to its high flammability [4], so the addition of CO_2_ to propane reduces the risk of flammability and cost. The use of propane together with CO_2_ also contributes to reducing the critical pressure of this system, which directly affects mass transfer due to the changes that occur in diffusivity, density and viscosity [5]. Furthermore, the combination of these solvents can increase the recovery of compounds; for example, γ-tocopherol [6] and squalene [7]. The use of SFE with propane and CO_2_ is an alternative when extracting nonpolar and moderately polar compounds, mainly the lipophilic fractions, is desired, resulting in a co-product rich in phenolic compounds. Furthermore, it is advantageous to use SFE in sequential processes, since in the depressurization stage it can cause the rupture of the cell walls [8], which facilitates the penetration of the solvent into the solid matrix in the subsequent stage.

The choice of extracting the leaves first using the SFE technique with nonpolar solvents is interesting with respect to removing the lipophilic fraction from the plant matrix, in addition to increasing the contact area between solute and solvent, facilitating the removal of phenolic compounds in the next step [8]. When this co-product is subjected to extraction with polar solvents, such as hydroethanolic solutions, the polar phenolic fraction of the plant matrix is obtained [9,10], and the PLE technique can be applied in this case. The technique referred to here operates using solvents in the liquid state; thus, the pressure and temperature variables are chosen in order to favor a reduction in the surface tension and viscosity of the solvent and promote an increase in the solubility of the compounds. In this way, the penetration of the solvent into the solid matrix occurs more easily [11,12].

*Pereskia aculeata* is an unconventional food plant, popularly known as ora-pro-nobis (OPN), which has been explored due to its composition—mainly its leaves—being attractive to several industrial niches due to its high protein, vitamin and fiber content. Additionally, the extracts obtained from these leaves stand out due to their antioxidant potential [13,14], antibiotic potential [15], and antinociceptive and anti-inflammatory activity [16,17]. The strategy for sequential extraction of compounds from OPN leaves has already been evaluated. Torres et al. [8] applied different extraction techniques using high pressure in sequence, namely SFE with CO_2_ and PLE with ethanol and water (separately). Torres et al. [18] implemented the following sequence of extraction methods: SFE with CO_2_, gas-expanded liquid extraction using CO_2_:ethanol, PLE with ethanol and, finally, subcritical water extraction.

In this context, the present investigation stands out for its use of a combination of techniques with solvents not yet explored, with the aim of obtaining extracts with different compositions. For this purpose, we explored propane and propane + CO_2_ in the SFE step, followed by the use of hydroethanolic solvent in PLE. The choice to implement this sequential extraction configuration becomes strategic, since the first extract will be rich in nonpolar compounds, while the extracts obtained by PLE will be rich in phenolic compounds. Additionally, a greater number of compounds can be obtained from the matrix by this methodology, and in the end both extracts can be combined.

## 2. Materials and Methods

### 2.1. Sample Acquisition and Preparation

The leaves of OPN, registered under number UNOP 10803 in the herbarium of the State University of Western Paraná (Cascavel, PR, Brazil), were collected in Toledo (PR, Brazil) (24°44′33.81″ S, 53°44′57.95″ W) in February 2024. The leaves (mature, ~10 cm) were collected in the morning, avoiding direct sunlight, according to instructions from Rodrigues [19]. To ensure sample homogeneity and traceability, the collection was performed only once. Figure S1 (Supplementary Material) shows a photo of the OPN leaves.

The leaves used were mainly young and were dried at 55 °C for 72 h according to method number 950.46 (AOAC, 2016) in an oven with air circulation (New Lab, model N1040, Piracicaba, SP, Brazil). The dried leaves, with a moisture content of 13.18 ± 1.08 wt%, were triturated using a blender (Spolu Attak, Model SPL-037, Itajobi, SP, Brazil), sieved (Tyler series sieves, 12 to 60 mesh) and stored in a freezer (−5 °C) until use. For the extractions, the fraction retained at 25 mesh (particle diameter of 710 μm) was used to ensure consistent particle size.

### 2.2. Materials

Propane (White Martins, 99.9%), carbon dioxide (Linde, 99.9%), ethanol (99.8% purity, Dinâmica, Indaiatuba, SP, Brazil) and reverse osmosis water (Evolution RO0310, Permutation) were used for the extractions.

In the subsequent characterization steps, the following materials were used: ethanol (99.8% purity, Panreac, Castellar del Vallès, BCN, Spain), Folin–Ciocalteu (Dinâmica), sodium carbonate (Dinâmica), 2,2-difenil-1-picrilhidrazil (DPPH•) (Sigma-Aldrich, 95% purity, Saint Louis, MI, USA), (±)-6-hydroxy-2,5,7,8-tetramethylchroman-2-carboxylic acid (Sigma-Aldrich, 97% purity), chromatographic standards of squalene (Sigma-Aldrich, 99.9% purity), octacosanol (Sigma-Aldrich, 99.9% purity), β-sitosterol (Sigma-Aldrich, 95% purity), α-tocopherol (Sigma-Aldrich, 99.9% purity) and gallic acid (Sigma-Aldrich, 99.9% purity), methanol (purity of ≥99.9%, Panreac) and acetic acid (Neon, Suzano, SP, Brazil).

The analysis of phenolic compounds by HPLC was performed using HPLC-grade methanol (Honeywell Riedel-de Haen, Seelze, Germany), ultrapure water (Milli-Q ultrapure water, Millipore, Burlington, MA, USA), formic acid (Sigma-Aldrich) and analytical standards of phenolic compounds. Gallic, 4-hydroxybenzoic, caffeic, chlorogenic, *p*-coumaric and ferulic acids, along with quercetin, were purchased from Sigma-Aldrich. Vanillic acid was purchased from Fluka, as were Rutin and malic acid and protocatechuic acid. The purity of all reference standards ranged from 95% to 99.3%.

For the analysis of crude protein, the following reagents were used: copper sulfate (Exodus Científica, Sumaré, SP, Brazil), sodium selenite (Exodus Científica), sodium sulfate (Dinâmica), sodium hydroxide (Neon), sulfuric acid (Neon) and boric acid (Exodus).

### 2.3. Extraction

**Figure 1:**
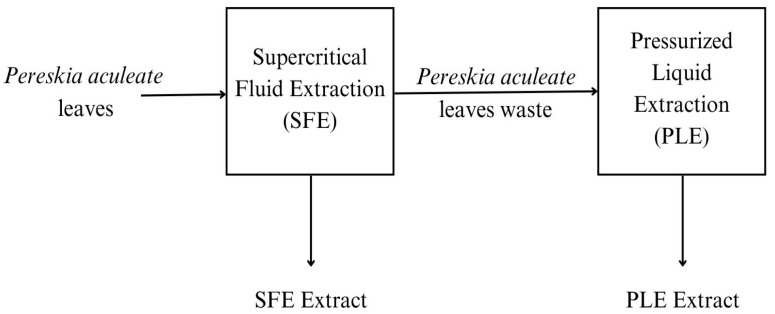
Experimental extraction processes.

The active compounds were obtained by two high-pressure methods (Figure 1): Supercritical Fluid Extraction (SFE) for the extraction of nonpolar compounds and Pressurized Liquid Extraction (PLE) to extract mainly polar compounds. In PLE, the biomass residue retained in the extractor from the SFE stage was used.

SFE was performed using an experimental apparatus and procedure detailed by Iwassa et al. [6]; as well, the extraction conditions were defined based on this study. These conditions were selected because they provided greater removal of oil, phytosterols and tocopherols. The temperature was kept fixed at 40 °C and pressures of 20 bar, 100 bar and 120 bar were adopted using propane, CO_2_ + propane (45% propane) and CO_2_ + propane (40% propane), respectively. In all tests, the extractor was filled with 15 g of OPN leaves, the solvent flow was maintained at 1.5 mL/min using a syringe pump (Teledyne ISCO, Model 500D, Lincoln, NE, USA) and the extraction time was 60 min.

The PLE was conducted following the procedure described by Passos et al. [20]. In each test, the extractor was filled with ~2 g of the SFE coproduct, and the operating conditions were 100 °C, 50 bar, hydroalcoholic solvent with 75% (*v*/*v*) ethanol, a solvent flow rate of 2 mL min^−1^ (high-pressure liquid pump, Waters, 515 model), and a static and dynamic time of 10 and 15 min, respectively. The dry extract was obtained by evaporating the solvent using a rotary evaporator (IKA rotary vacuum evaporator, RV 10) at 50 °C.

The extraction yield for both techniques was calculated considering the ratio between the mass of extract obtained in the step and the initial mass of material fed into the extractor (on a dry basis).

### 2.4. Characterization Analyses

The total phenolic compound (TPC) content was determined by the Folin–Ciocalteu method [21] and the antioxidant potential was evaluated by the DPPH• method [22]. The absorbance of the solutions was determined in a spectrophotometer (Kasuaki, DR-200BS-NM-BI, Wuxi, China).

The analysis of the phenolic compound profile was conducted on an ultra-high performance liquid chromatograph coupled to a triple-quadrupole mass spectrometer (UHPLC-MS/MS, Waters ZTQD Acquity LCMS, Milford, MA, USA), using a Waters ZTQD LCMS Acquity model equipped with an electrospray ionization (ESI) source, operated in positive and negative mode. The quantification of the compounds was performed from standard curves obtained from the analytical standards of the compounds in the concentration range of 0.05 to 20 mg/L (R2 > 0.99). Other analytical conditions and the detection limits of the method are presented by Rodrigues et al. [23].

Identification of compounds by gas chromatography was conducted in a gas chromatograph (GC-MS QP2010 SE, Shimadzu, Japan, Tokyo) using a flame ionization detector, as reported by Passos et al. [20] and Rosa et al. [24]. Protein content was quantified based on the Kjeldahl method [25].

The experiments and analyses were performed in duplicate (n = 4). The data obtained were subjected to analysis of variance (ANOVA) and application of the Tukey test, and a significant difference was considered with a *p*-value < 0.05.

## 3. Results

### 3.1. Extraction Yield

Table 1 presents the results obtained regarding the performance of the applied techniques. In the steps applying SFE, the highest EY was obtained using only propane at a lower operating pressure, with a value 50% higher than the other conditions. This may occur because the use of CO_2_ can cause instability in the liquid phase, hindering the miscibility of the pressurized fluid and extract [7,26]. Furthermore, propane is more efficient at removing the nonpolar fraction than CO_2_, as previously shown [3,6], since these compounds have greater solubility in propane at lower pressures.

The lower yield of SFE compared to PLE is related to the greater selectivity of SFE, which preferentially extracts nonpolar or moderately polar compounds, such as carotenoids, tocopherols and fatty acids [5]. The greater efficiency of PLE results from the affinity of polar compounds, such as phenolics, for the hydroalcoholic solvent, which, being less selective, allows for the extraction of a wider range of compounds [27]. When comparing SFE + PLE with PLE alone, it is noted that the yield differed only from test 3 (SFE/propane). This result can be attributed to the solvent composition and pressure used in SFE [6], which resulted in a greater recovery of compounds from the OPN leaves, affecting the recovery of compounds in the next step.

### 3.2. Extracts Characterization

Table 2 presents characterization data from the extracts obtained, showing that the recovery of phenolic compounds was more efficient in PLE, due to the polarity of the solvent used and because the SFE process has a benefit in terms of the contact of the solvent with the leaves due to depressurization. When comparing sequential extraction with isolated PLE (assay 4), there was a difference in the recovery of TPC only for assay 1. Vardenaga et al. [28] verified a benefit in terms of the recovery of phenolic compounds from granadilla residue by PLE, after the removal of lipophilic compounds using SFE/CO_2_, and attributed this benefit to the desorption of TPC being facilitated after the removal of lipophilic compounds. Krümmel et al. [11] observed the same behavior with respect to an increase in obtaining TPC by PLE after the SFE step due to the removal of the nonpolar fraction from the matrix, further indicating that this increase may be due to the majority of phenolic compounds being polar and therefore having greater affinity with ethanol.

The difference between the techniques and solvents used can be observed in the profile of the phenolic compounds of the samples obtained. SFE presented a lower number of phenolic compounds compared to PLE. This can be attributed to the polarity of the solvents used (propane and CO_2_), both nonpolar, which have a greater affinity for lipophilic compounds. The greater recovery of phenolics by polar solvent was also evidenced by Torre et al. [8] and Mazutti et al. [12].

The extracts obtained by SFE presented the lowest values of antioxidant potential (DPPH•), consistent with the lowest values of TPC content. The sequential extraction process made it possible to obtain extracts with greater antioxidant potential compared to SFE and PLE. This can be attributed to the removal of lipophilic compounds in the first extraction, acting as a purification step for the next step, by PLE using hydroalcoholic solvent, which is recognized for its affinity with these compounds. In addition, the combination of solvent factors and temperature and pressure conditions, in synergy, benefit the recovery of polar compounds. When comparing SFE with PLE, the same behavior of TPC was noted; that is, extraction by PLE with ethanol solvent (75%) is more efficient for the recovery of compounds with antioxidant potential. Although isolated PLE (run 4) provided extract with higher TPC content values, it presented lower antioxidant activity than those obtained from the combination of SFE + PLE, which indicates that other unidentified compounds may contribute to the antioxidant potential of the SFE + PLE extract or the PLE extract presents compounds that may reduce this potential.

The phenolic compounds quantified in the extracts produced by PLE are shown in Table 3. Among the quantified compounds, those with the highest levels were caffeic, nicotinic, protocatechuic and *p*-coumaric acids, with caffeic and nicotinic acids representing 45% of the content of identified phenolic compounds. From the data in this table, it can be seen that sequential extraction was more efficient in removing these compounds compared to isolated PLE (run 4).

Caffeic acid is recognized in the literature for its photoprotective effect, being able to penetrate the deepest layers of the skin, in addition to being an antioxidant [29]. Nicotinic acid has a wide variety of applications, being commercially applied for the treatment of hyperlipidemia and hypertriglyceridemia [30].

Table 4 presents the levels of lipophilic compounds quantified in the extracts by SFE and PLE, in which it is shown that SFE promoted a greater recovery of these compounds when compared to PLE alone. This can be attributed to the solvents used and the molecular structure of the compounds. Among these compounds, squalene and α-tocopherol were identified by Torres et al. [17] in extracts of OPN leaves.

The recovery of lipophilic compounds benefits from the the use of less polar solvents [31]. In the present study, this evidence was confirmed, as the use of SFE with propane + CO_2_ was efficient in recovering these compounds. Squalene was quantified in the extract obtained by PLE (assay 4); however, the concentration was ~3× lower. This fact can be attributed to the polarity of the solvent and the thermosensitivity of the compound, as PLE was performed at 100 °C, while the SFE with the highest squalene content occurred at 40 °C. Santos et al. [32] applied SFE at 40, 60 and 80 °C to passion fruit residue and established the best condition for squalene extraction at 40 °C, noting a decrease of 42.77% and 46.81% when increasing the temperature to 60 and 80 °C, respectively. Abrantes et al. [7] also noted that SFE with propane + CO_2_ provided 4.5 times higher squalene content compared to the use of propane. The authors attributed this to the solvation power of the solvent mixture, which resulted in a greater affinity for squalene.

All compounds evaluated showed higher recovery when SFE was used. In addition to the influence of the solvent, the influence of temperature on the extraction was observed. When compared to sequential PLE extraction, the recovery of octacosanol was ~15× higher and in isolated PLE it was ~7× higher. The same behavior was noted for α-tocopherol, in which SFE presented a content 2× higher than PLE (sequential and isolated). To obtain the greatest amount of lipophilic compounds, the use of low temperatures is the most indicated to avoid degradation of the compounds. The synergy between temperature, pressure and solvent composition promoted an attractive composition of lipophilic compounds, with a high content, compared to the use of PLE. The use of mild temperature (40 °C) benefited the recovery of lipophilic compounds, as they are thermosensitive.

It is worth noting that, based on the data in Table 3 and Table 4, it can be seen that the extracts obtained from the combination of SFE + PLE have a composition that surpasses that of the application of PLE alone. Thus, the adopted strategy made it possible to obtain a higher-quality extract (considering the compounds evaluated), since there was greater recovery of the compounds available in the plant matrix.

Table 5 shows the protein content in the residual biomass from the sequential extraction process and in the leaf. OPN leaves are recognized for their high protein content, which can reach up to 28% (dry basis) [33]. In the present study, the residual biomass from the extraction process presented approximately 24% protein, indicating that the amount of protein was maintained even after the use of pressurized processes. Aiming at a sustainable process and maximum use of the raw material (OPN leaf), the residual biomass could be used for the production of protein concentrates and nutritional enrichment of foods, for example.

## 4. Conclusions

The present study aimed to enhance the value of OPN leaves through the use of pressurized extraction processes (SFE and PLE) applying the biorefinery concept. This strategy proved to be efficient for obtaining OPN extracts, considering the yield obtained in each technique and the composition of the extracts. In SFE, extracts rich in lipophilic compounds were obtained, while in PLE, extracts rich in phenolic acids and with antioxidant potential were obtained, which was a benefit arising from the removal of lipophilic compounds and the depressurization that occurred in SFE. As an overview, it can be seen that the combination of extracts obtained from each technique results in a differentiated product due to the compounds identified. Thus, the application of biorefinery for the production of extracts from OPN leaves has proven to be promising, especially considering its application in nutraceutical, pharmaceutical and food industries. Additionally, the conditions defined in this study can be applied to recover the analyzed compounds from other matrices.

## Figures and Tables

**Table 1:** Results obtained in terms of extraction yield (EY) by Supercritical Fluid Extraction (SFE) and Pressurized Liquid xtraction (PLE).

Assay^-^	Extraction Type	Step	T (°C)	P (bar)	EY (wt%)
1	SFE/Propane + CO_2_	1	40	120	1.1 ± 0.03 ^bc^
	PLE	2	100	50	19.2 ± 0.8 ^AB^
2	SFE/Propane + CO_2_	1	40	100	1.3 ± 0.2 ^b^
	PLE	2	100	50	18.9 ± 1.1 ^AB^
3	SFE/Propane	1	40	20	1.9 ± 0.2 ^a^
	PLE	2	100	50	16.5 ± 0.2 ^B^
4 ^1^	PLE	-	100	50	21.1 ± 0.9 ^A^

T: temperature; P: pressure. ^1^ Assay from Passos et al. [20]. Different letters represent statistical differences between the responses at a 5% confidence level to the same extraction technique (lowercase letters represent SFE and capital letters represent PLE).

**Table 2:** Characterization of the dry extract obtained from Supercritical Fluid Extraction (SFE) and Pressurized Liquid Extraction (PLE) in relation to total phenolic compound (TPC) content, identified phenolic compounds and antioxidant activity.

Property		Assay ^1^						
		1		2		3		4
		SFE	PLE	SFE	PLE	SFE	PLE	PLE
TPC (mg_GAE_ g dry extract ^1^)		21.5 ± 0.5 ^c^	34.0 ± 0.6 ^b^	15.9 ± 0.5 ^d^	40.7 ± 2.2 ^a^	20.7 ± 0.3 ^c^	37.5 ± 1.2 ^ab^	40.1 ± 0.8 ^ab^
Phenolic acid ^2^	Gallic	nd	+	+	+	nd	+	+
	4-Hydroxybenzoic	+	+	+	+	+	+	+
	*p*-Coumaric	−	+	−	+	−	+	+
	Ferulic	+	+	+	+	+	+	+
	Siringic	+	+	+	+	+	+	+
	Malic	+	+	+	+	+	+	+
	Protocatechuic	−	+	−	+	−	+	+
	Vanillic	+	+	+	+	+	+	+
	Caffeic	+	+	+	+	−	+	+
Flavonoid ^2^	Quercetin	−	+	−	+	−	+	+
	Rutina	+	+	+	+	+	+	+
Nicotinic acid ^2^		+	+	+	+	+	+	+
DPPH• (µmol_TE_ g dry extract^−1^)		87.8 ± 3.0 ^c^	243.9 ± 6.4 ^a^	86.7 ± 5.3 ^c^	247.7 ± 2.9 ^a^	90.5 ± 2.3 ^c^	235.3 ± 5.3 ^a^	155.7 ± 1.7 ^b^

^1^ as Table 1. GAE: gallic acid equivalent. ^2^ (+) means presence and (−) means absence. nd: not detected. Different letters (on each line) represent statistical differences between the responses at a 5% confidence level.

**Table 3:** Quantification of phenolic compounds in dry extract obtained by Pressurized Liquid Extraction (PLE).

Compound (mg per 100 g Dry Extract)		Assay from PLE ^1^			
		1	2	3	4
Phenolic acids	Gallic	0.7 ± 0.2 ^a^	1.4 ± 0.1 ^a^	0.7 ± 0.1 ^a^	0.5 ± 0.1 ^a^
	*p*-Coumaric	8.7 ± 1.1 ^a^	9.9 ± 1.0 ^a^	9.4 ± 1.3 ^a^	8.3 ± 0.6 ^a^
	Ferulic	0.5 ± 0.1 ^a^	0.6 ± 0.05 ^a^	0.5 ± 0.05 ^a^	0.6 ± 0.1 ^a^
	Siringic	3.3 ± 0.6 ^a^	2.2 ± 0.3 ^a^	3.8 ± 0.2 ^b^	1.6 ± 0.1 ^a^
	Protocatechuic	10.6 ± 0.9 ^b^	17.3 ± 0.7 ^a^	10.3 ± 0.9 ^b^	7.1 ± 0.5 ^c^
	Caffeic	31.1 ± 1.0 ^a^	32.6 ± 0.8 ^a^	30.5 ± 0.5 ^a^	29.0 ± 0.7 ^a^
Flavonoids	Quercetin	0.5 ± 0.01 ^a^	0.7 ± 0.1 ^a^	0.6 ± 0.1 ^a^	0.5 ± 0.1 ^a^
Nicotinic acid		11.6 ± 0.5 ^a^	13.8 ± 0.2 ^a^	12.5 ± 0.4 ^a^	8.3 ± 0.1 ^b^
Total		67.0 ± 4.4 ^b^	78.5 ± 3.2 ^a^	68.4 ± 3.7 ^b^	55.9 ± 2.3 ^c^

^1^ as Table 1. Different letters (on each line) represent statistical differences between the responses at a 5% confidence level.

**Table 4:** Quantification of lipophilic compounds in the dry extract obtained from Supercritical Fluid Extraction (SFE) and Pressurized Liquid Extraction (PLE).

Compounds Quantified by GC-FID (mg/100 g_extract_)	Assay ^1^						
	1		2		3		4
	SFE	PLE	SFE	PLE	SFE	PLE	PLE
Squalene	276.6 ± 2.8 ^a^	nd	187.1 ± 0.2 ^b^	nd	167.3 ± 9.9 ^b^	nd	95.0 ± 3.0 ^c^
Octacosanol	8517.5 ± 861.7 ^a^	664.8 ± 35.4 ^b^	9804.6 ± 442.6 ^a^	650.9 ± 8.9 ^b^	8687.1 ± 743.5 ^a^	427.8 ± 3.6 ^c^	1157.3 ± 74.9 ^d^
α-tocopherol	271.6 ± 27.2 ^ab^	49.7 ± 7.6 ^d^	173.3 ± 2.4 ^bc^	73.9 ± 9.96 ^d^	286.7 ± 38.5 ^a^	64.4 ± 4.2 ^d^	110.1 ± 11.2 ^d^
β-sitosterol	1049.1 ± 52.8 ^a^	369.1 ± 6.4 ^b^	1025.0 ± 47.1 ^a^	327.4 ± 2.2 ^b^	1125.3 ± 33.3 ^a^	292.5 ± 21.6 ^b^	421.6 ± 10.7 ^b^

^1^ as Table 1. Different letters (on each line) represent statistical differences between the responses at a 5% confidence level.

**Table 5:** Quantification of proteins and fibers.

Compound (Dry Basis)	Leaf	Assay ^1^			
		1	2	3	4
Protein (g/100 g)	24.0 ± 0.6 ^a^	22.4 ± 0.5 ^a^	23.1 ± 0.5 ^a^	23.7 ± 0.4 ^a^	23.2 ± 0.07 ^a^

^1^ as Table 1. Different letters (on each line) represent statistical differences between the responses at a 5% confidence level.

## Data Availability

The data presented in this study are available in the present article.

## Supplementary Materials

The following supporting information can be downloaded at https://www.mdpi.com/article/10.3390/plants14131956/s1: Figure S1, Photos of the Pereskia aculeata leaves.
