# Supplementary figures and images for: Application of Sequential Extraction Using Pressurized Fluids to Obtain Compounds from Pereskia aculeata Leaves
Source: Plants (Basel). 2025 Jun 26;14(13):1956. doi: 10.3390/plants14131956 (PMC12251725; doi:10.3390/plants14131956)

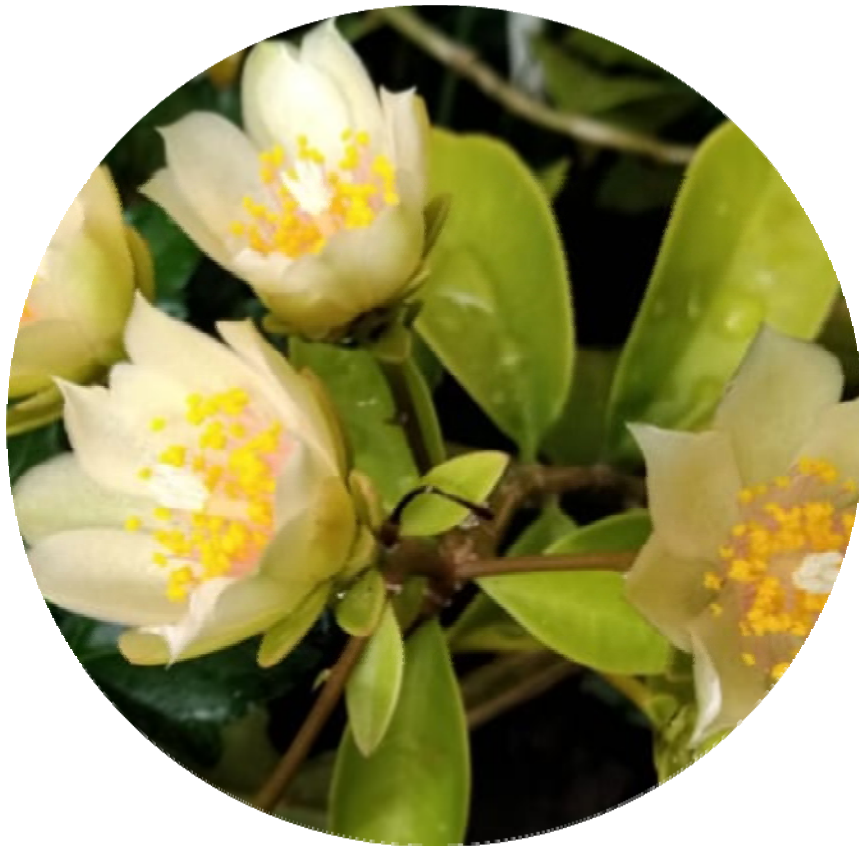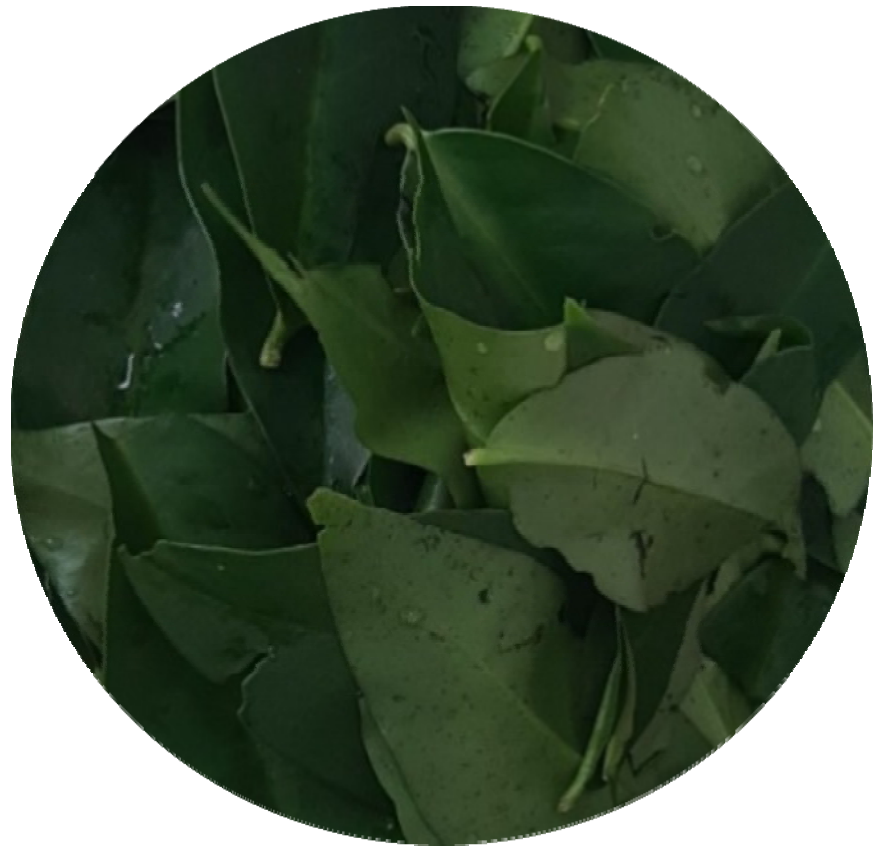

**Figure S1.** Photos of the *Pereskia aculeata* leaves.

Supplement: Supplementary file 1 [file plants-14-01956-s001.zip › plants-3644515-supplementary.pdf]
